# Increased expression of musashi 1 on breast cancer cells has implication to understand dormancy and survival in bone marrow

**DOI:** 10.18632/aging.204620

**Published:** 2023-03-29

**Authors:** George R. Nahas, Lauren S. Sherman, Garima Sinha, Markos H. El Far, Andrew Petryna, Steven M. Munoz, Kimberly A. Silverio, Maran Shaker, Pujan Neopane, Veronica Mariotti, Pranela Rameshwar

**Affiliations:** 1 Department of Medicine, Hematology-Oncology, Rutgers New Jersey Medical School, Newark, NJ 07103, USA; 2 Rutgers School of Graduate Studies at New Jersey Medical School, Newark, NJ 07103, USA

**Keywords:** cancer stem cell, breast cancer, musashi 1, bone marrow, dormancy

## Abstract

Breast cancer (BC) stem cells (CSCs) resist treatment and can exist as dormant cells in tissues such as the bone marrow (BM). Years before clinical diagnosis, BC cells (BCCs) could migrate from the primary site where the BM niche cells facilitate dedifferentiation into CSCs. Additionally, dedifferentiation could occur by cell autonomous methods. Here we studied the role of Msi 1, a RNA-binding protein, Musashi I (Msi 1). We also analyzed its relationship with the T-cell inhibitory molecule programmed death-ligand 1 (PD-L1) in CSCs. PD-L1 is an immune checkpoint that is a target in immune therapy for cancers. Msi 1 can support BCC growth through stabilization of oncogenic transcripts and modulation of stem cell-related gene expression. We reported on a role for Msi 1 to maintain CSCs. This seemed to occur by the differentiation of CSCs to more matured BCCs. This correlated with increased transition from cycling quiescence and reduced expression of stem cell-linked genes. CSCs co-expressed Msi 1 and PD-L1. Msi 1 knockdown led to a significant decrease in CSCs with undetectable PD-L1. This study has implications for Msi 1 as a therapeutic target, in combination with immune checkpoint inhibitor. Such treatment could also prevent dedifferentiation of breast cancer to CSCs, and to reverse tumor dormancy. The proposed combined treatment might be appropriate for other solid tumors.

## INTRODUCTION

The two human musashi genes (*Msi 1 and Msi 2*) share 75% homology [1]. Msi 1 and 2 are mostly reported as RNA-binding proteins [1]. Msi, along with the Hu (Elav) family, are well-described RNA-binding proteins [2, 3]. Msi 1 regulates several regulatory pathways in cell development [4]. Msi 1 regulates the translation of transcription factors and cell cycle proteins to affect downstream pathways to modulate gene expression [5].

Early studies identified Msi 1 in the brain, and ascribed Msi 2 as a regulator of hematopoietic stem cell function as well as support for hematological and solid tumors [6, 7]. There is interest in Msi 2 as a target in drug development [1]. Msi 1 promotes breast cancer (BC) cell (BCC) growth [8]. This role is partly explained by the interaction between Msi 1 and the 3’ untranslated region (3’ UTR) of TAC 1 mRNA, which stabilizes the transcript [8]. Msi 1 competed with suppressive miRNAs for the same site on the UTR region of TAC 1 mRNA [8]. Since TAC 1 peptides and their receptors are oncogenic, these findings provide a partial mechanism by which Msi 1 could mediate oncogenic function [9–14]. Importantly, the experimental evidence indicated a role for Msi 1 in maintaining cancer stem cells (CSCs), indicating a key role for Msi 1 during various phases of tumorigenesis [8].

Msi 1 could support CSCs through its ability to regulate the expression of genes involved in cell renewal and differentiation, such as Notch [15]. Numb negatively regulates Notch to suppress the expression of Notch-targeted genes [16]. Numb recruits the ubiquitination system to Notch receptor, which prevents nuclear translocation of the intracellular domain (NCID) to negatively affect the expression of Notch-targeted genes [17–19]. Msi 1 supports Notch 1 expression during asymmetric division of stem cells by blocking Numb translation [20]. In other mechanism, Notch 1 regulates *Hes 1* expression to maintain stem cell functions [20]. Notch 1-Msi 1 link is relevant to the function of healthy and cancer stem cells, including glioblastoma stem cells [5, 21, 22]. Thus, insights into the role of Msi 1 in cancer cells will provide information on its role at different phases of cancer – metastatic and during the period of remission when the tumor is in a dormant phase [23]. BCC dormant cells have been reported to adapt stemness [24].

In light of the growing interest of Msi 1 as a potential cancer target, it would be interesting to initiate studies to determine if this molecule is linked to the expression of the T-cell inhibitory molecule programmed death-ligand 1 (PD-L1) and if so, whether such regulation is limited to particular BCC subset. Interaction between PD-L1 on cancer cells and PD-1 on T-cells serves as immune checkpoint. PD-L1 is expressed on triple negative BC and other cancers [25, 26]. This leads to immune checkpoint inhibitors as targets for cancer to enhance the endogenous immune system [26]. More importantly, the literature reported on Msi 1 and PD-L1 expression on BC CSCs [27]. However, it is unclear if both Msi 1 and PD-L1 are co-expressed on CSCs, and if so, how this could be interrogated for future cancer treatment.

PD-L1 expression in triple negative BC is mediated by the PTEN-PI3K-AKT pathway [25]. This occurs by PD-1 induced signaling in T cells to activate PI3K and AKT [28]. As compared to the other BCC subsets, we now found higher levels of PD-L1 on CSCs, and further showed that this increase correlated with enhanced Msi-1 [8, 15]. We further showed that Msi 1 is involved in the expression of PD-L1 in CSCs. This role correlated with Msi 1 involvement in regulating the expression of stem cell-associated genes in BCCs. We recapitulated a situation in which Msi 1, if targeted with shRNA, causes CSC loss, apparently by differentiation to more mature BCCs while supporting cell survival by increased Bcl 2. CSCs, which are cycling quiescent cells, will proliferate upon differentiation to other relatively more mature subsets. Thus, Msi 1 knockdown BCCs, which induced differentiation, showed evidence of transition from quiescence into cycling cells. However, we noted that continued cycling was halted by an increase in p-PTEN with concomitant decrease of the PI3K-pAKT axis. The latter explains the noted decrease in proliferation, despite differentiated CSCs in Msi 1 knockdown cells. The potential for Msi 1 as a therapeutic target, in combination with immune checkpoint inhibitor, as well as the implication to prevent and target dormant CSCs are discussed.

## MATERIALS AND METHODS

### Reagents

DMEM was purchased from Life Technology (Grand Island, NY), fetal calf sera (premium) from Hyclone Laboratories (Logan, UT), lipofectamine 3000 transfection reagent, M-PER Mammalian Protein Extraction Reagent, SuperSignal West Femto Maximum Sensitivity Substrate reagent, and Restore Western Blot Stripping Buffer from Thermo Scientific (Rockford IL), bovine serum albumin (BSA) from Sigma Aldrich (St Louis, MO), and effectene reagent from Qiagen (Germantown, MD).

### Antibodies

The following antibodies were purchased from Santa Cruz Biotechnology Inc. (Santa Cruz, CA): rabbit anti-p53, rabbit anti-Bcl2, rabbit anti-Cdk4, rabbit anti-BCL2, and rabbit anti-p15. Goat anti-p21 was purchased from R&D Systems (Minneapolis, MN), goat anti-Oct4a, and rabbit anti-Msi-1 from Abcam (Cambridge, MA); mouse anti-Hes1 from NovusBio (Centennial, Colorado); rabbit anti-p16, rabbit anti-phospho-PTEN, mouse anti-Rb, rabbit anti-phospho-PDK1, rabbit anti-PTEN, rabbit anti-PD-L1 and mouse anti-Cyclin E from Cell Signaling Technology (Massachusetts, USA). Cell Signaling kindly provided rabbit anti-phospho AKT as part of a sample kit. Mouse anti-PI3K was purchased from Millipore (St. Louis, MO), mouse anti-β-actin from Sigma-Aldrich, and mouse anti rabbit IgG-Alexafluor 647 from Invitrogen (Thermo Fisher Scientific, Waltham, MA).

### Vectors

pEGFP1-Oct3/4, which contains the 5’ regulatory region of *Oct4a*, upstream of green fluorescence protein (GFP), was generously provided by Dr. Wei Cui (Imperial College London, UK) [29]. The use of this vector to prepare stable BCCs has been described [24]. In this study, MDA-MB-231, stably transfected with pEGFP1-Oct3/4 was maintained in G418 as described [24]. GFP intensity was correlated with the relative maturing in which the CSCs expressed the most intense GFP intensity [24]. Thus, BCC subsets were based on the relative GFP intensity.

pRFP-C-RS with Msi1 shRNA insert (pRFP-C-RS-Msi1) was purchased from OriGene (Rockville, MD); pHes1-Luc, which contained the *Hes1* 5’ regulatory region, was kindly provided by Dr. Ryoichiro Kageyama, Kyoto University (Kyoto, Japan) [30]. pOct4a-Luc was prepared by excising the 5’ regulatory region of pEGFP1-Oct3/4 with *EcoRI* and *BamH1* from pEGFP1-Oct3/4 and then inserted into linearized (*Xho1*) pGL3 (Promega, Madison, WI) [24]. The fragment was first blunt-ended and then ligated into pGL3. The latter used T4 DNA ligase (New England Biolabs, Ipswich, MA) at vector:insert molar ratio of 1:3. The procedure used reagents within the kit with overnight incubation at 16° C. The reaction was halted at 65° C for 10 mins. The ligated vector was used to transform supercompetent *E. coli*. The insert was confirmed by DNA sequencing.

### Cells

BCCs, MDA-MB-231 (highly invasive, basal-like), were purchased from American Type Culture Collection (ATCC). The cells were cultured as per ATCC instructions. BCCs, stably transfected with Oct4-GFP, were previously described [24]. Briefly, the cells were transfected with pEGFP1-Oct3/4 and then selected with neomycin. Msi 1 knockdown (KD) was accomplished by transfecting the BCCs with a pRFP-C-RS vector containing Msi 1 shRNA as described [8].

### Sorting of BCC subsets

BC cell subsets were sorted on the FACSDiva (BD Biosciences, San Jose, CA), as described [24]. Briefly, BCCs, stably transfected with pEGFP1-Oct4a, were selected, based on relative GFP expression. The top 5% GFP (Oct4a^hi^) cells contained CSCs [24]. This was followed by Oct4a^med^ and Oct4a^lo^ [31].

### Flow cytometry

BCCs (5x10^6^), stably transfected with pOct4a-GFP, were fixed with 1 ml of 3.7% formaldehyde (diluted in 1x PBS) for 15 min at room temperature. After this, the cells were permeabilized for 30 min at room temperature using 1 ml of 0.2% Triton-X 100 (diluted in PBS). The cells were washed twice with 2 ml of 0.5% BSA, diluted in 1x PBS (incubation buffer) and then resuspended at 1:1 ratio of methanol:PBS. The cells were placed on ice for 10 mins. After this, the cells were pelleted, washed twice with incubation buffer and then resuspended at 10^6^/150 μL of incubation buffer. The cells were labeled for Msi 1 (1/40 final dilution) and PD-L1 (1:100 final dilution). Parallel labeling controls included cells incubated with media or isotype. The cells were incubated at room temp for 60 min. After this, the cells were washed twice with incubation buffer. Msi 1 KD BCCs were labeled for PD-L1 with anti-PD-L1 at 1/100 dilution followed by secondary Alexafluor 647 antibody at 1/200 dilution, similar to other secondary antibodies, with 30 min incubation at room temperature in the dark. This was followed by two washes with incubation buffer. The cells were resuspended in 1% formaldehyde and then immediately analyzed on the FACSCalibur (Becton and Dickson). The data were analyzed with BD CellQuest.

### Western blot analyses

Cell pellets were lysed with MPER, as per manufacturer’s instruction. Protein levels within the extracts were quantified with Bradford reagents (BioRad (Hercules, CA). Proteins (15 μg) were electrophoresed on 10% SDS-PAGE gels at 125 V. The proteins were transferred to PVDF membrane at 100 V for 1.5 h followed by incubation with primary antibodies, all at 1/1000 final dilution. The primary antibodies were detected with HRP conjugated anti-IgG at 1/1000 final dilution. HRP was developed with SuperSignal West Femto Maximum Sensitivity Substrate and developed. Bands were detected on the ChemiDoc XRS+ system (BioRad, Hercules, CA). Membranes were stripped with Restore Western Blot Stripping Buffer prior to reprobing for additional proteins. The densities for the experimental bands were normalized to β-actin with UN-SCAN-IT densitometry software (Silk Scientific; Orem, UT). Normalization was done by dividing the band densities of each experimental band by its respective β-actin band density. The full membranes for the Western blots are included in the Supplementary Figures 1–3.

### Transfection and reporter gene assay

The reporter gene vectors, described above, were used in assays, as described [23]. Briefly, BCCs were co-transfected with pHes1-Luc, pOct4a-Luc or pCyclin D1-Luc and pβgal. Transfection used effectene reagent, as per manufacturer’s instruction. After 48 h, cell-free lysates were prepared using 1X Promega lysis buffer. The lysates were quantitated for total protein with the Bradford protein assay reagent (BioRad, Hercules, CA). Extracts (10 μg) were analyzed for luciferase activity and β-galactosidase (β-gal) using kits from Promega. The relative luminescence unit (RLU) for luciferase values was normalized with β-gal.

### RNA mRNA-protein prediction

The RNA-Binding potential of proteins was analyzed using the RPI-Seq software from Iowa State University (http://pridb.gdcb.iastate.edu/RPISeq/index.html). The software requires two query fields in plain-text format, the amino acid sequence of the protein and a nucleic acid sequence of the target. The software uses existing interaction databases to predict whether domains within the primary amino acid sequence can bind to the target nucleic acid string. The input of the amino acid sequences for relevant protein isoforms were selected from the National Library of Medicine’s NCBI website and were inserted into the protein sequence field of the RPI-Seq website. RNA transcripts were also sourced from the NLM NCBI database and were entered in plain text format into the RNA query field. The analysis was then run and a numerical value between 0.0 and 1.0 was generated based on the interaction probability [32, 33].

### Statistical analyses

Statistical data analyses were performed with analysis of variance and Tukey-Kramer multiple comparisons test. *P*<0.05 was considered significant.

## RESULTS

### Evolutionary conservation of Msi 1

The literature indicated that Msi 1 could regulate healthy and malignant stem cells (CSCs) [8, 15]. In order to gain insights into the biological role of Msi 1, we asked if this gene is evolutionary conserved. An open source software (Ensembl) indicated 93% average alignment of the Msi 1 sequence among 89 species of placental mammals, and an average of 95% alignment between human and 23 primates [34]. BlastP queries showed an average of 72% alignment of the primary amino acid Msi 1 sequence with the orthologous proteins of 5000 organisms [35]. Taken together, the conserved nature of Msi 1 strongly suggests a critical role in vital developmental systems. These analyses led us to focus on a role for Msi 1 in CSCs and to determine if Msi 1 is linked to immune checkpoint.

### Msi 1 levels in BCC subsets

**Figure 1:**
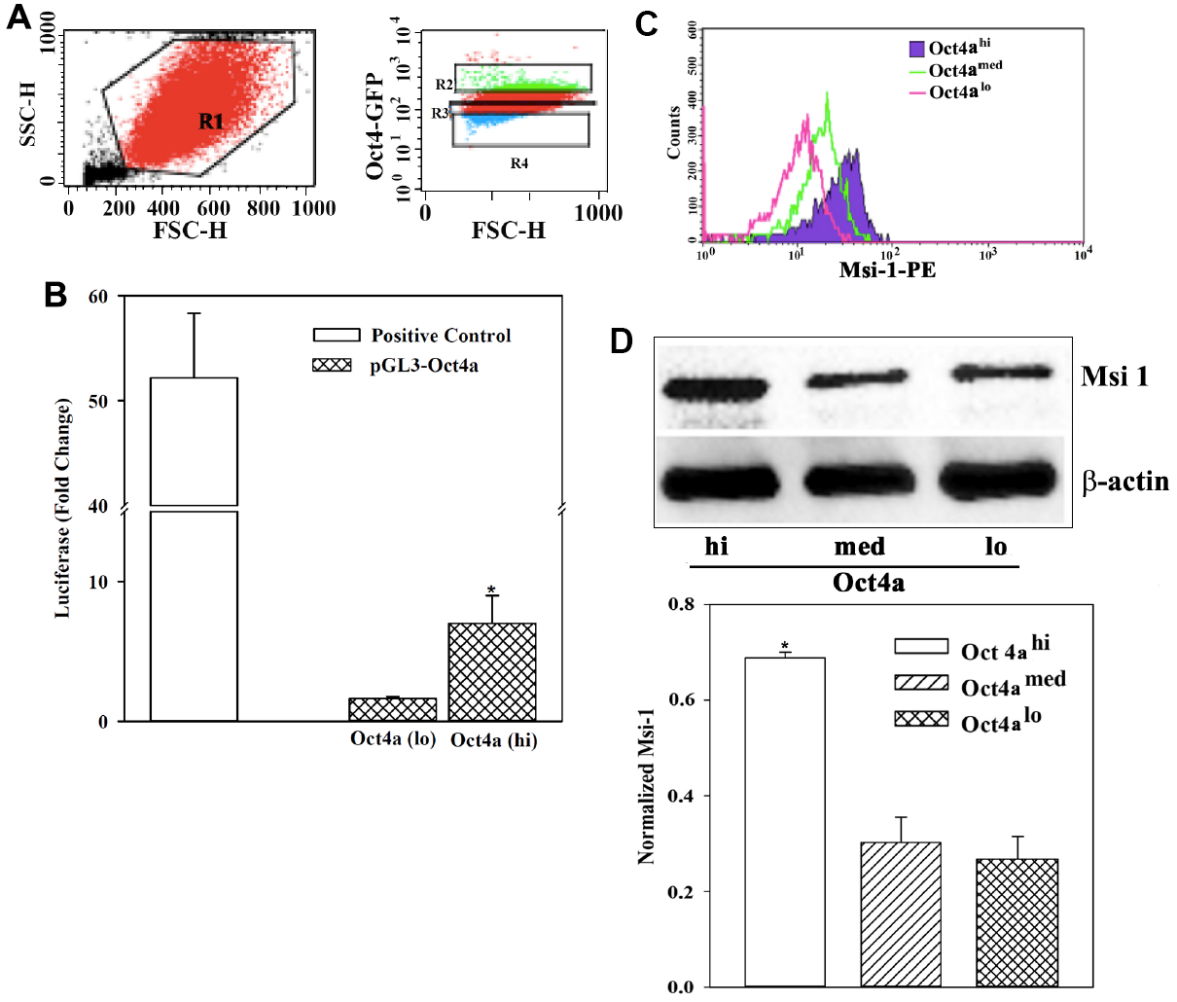
**Msi 1 expression in BCC subsets.** (**A**) Shown is the gating scheme for MDA-MB-231 cells, stably expressing pOct4a-GFP for different GFP intensities. (**B**) MDA-MB-231 cells with stable pOct4a-GFP were sorted for Oct4a^hi^ (CSCs) and Oct4a^lo^ cells. The cells were transfected with pGL3-Luc under the control of the upstream regulatory region of *Oct4a*. Control included unsorted BCCs transfected with pGL3-Luc under the control of the CMV promoter (open bar). The mean fold change of luciferase is presented for three independent experiments. * *p*<0.05 vs. Oct4a^lo^ BCCs. (**C**) Flow cytometry was performed for Msi 1 using the gating scheme shown in `A’. The histogram represents five different experiments. (**D**) MDA-MB-231 with pOct4a-GFP were sorted for Oct4a (hi) or (lo) subsets and their whole cell extracts were studied for Msi 1 by western blot. The normalized band densities (mean±SD) of three independent experiments below. * *p*<0.05 vs. Oct4a^med^ or Oct4a^lo^.

Msi 1 facilitated BCC growth, chemoresistance, and likely support CSCs [4, 8]. Previous studies have identified dormant BCCs in the bone marrow as CSCs [24]. Thus, an understanding of how Msi 1 regulates CSCs will contribute to the mechanisms by which BCCs survive as dormant cells in the bone marrow niche [23, 24]. We took advantage of our established model to discern different subsets of MDA-MB-231 with stable pOct4a-GFP. The intensity of GFP correlated with Oct4a levels with the subsets showing the highest GFP intensity as CSCs (Oct4a^hi^) [23, 24, 31] (Figure 1A). We further validated the correlation between GFP intensity and Oct4a expression using reporter gene studies. We transfected pOct4a-Luc into Oct4a^hi^ and Oct4a^lo^ BCCs. Technical control used MDA-MB-231 cells, transfected with pGL3-Luc under the control of CMV promoter [36]. Luciferase level was significantly (*p*<0.05) reduce in Oct4a^lo^ BCCs as compared to Oct4a^hi^ BCCs, indicating that Oct4a levels could be assessed with GFP intensity (Figure 1B).

Next, we studied BCC subsets for Msi 1 by intracellular labeling. Flow cytometry indicated a direct correlation between Msi 1 and GFP intensity (Figure 1C). The highest Msi 1 level was observed in Oct4a^hi^ BCCs, followed by Oct4a^med^ then Oct4a^lo^. Western blot for Msi 1 verified the flow cytometry outcome – The band with extracts from Oct4a^hi^ BCCs was the most intense, followed by Oct4a^med^, then Oct4a^lo^ MDA-MB-123 cells (Figure 1D). In summary, we showed a direct relationship between Msi 1 and the relative stemness of BCCs. The latter was based on a working BCC hierarchy [24, 31].

### Decreased CSCs in Msi 1 knockdown BCCs

**Figure 2:**
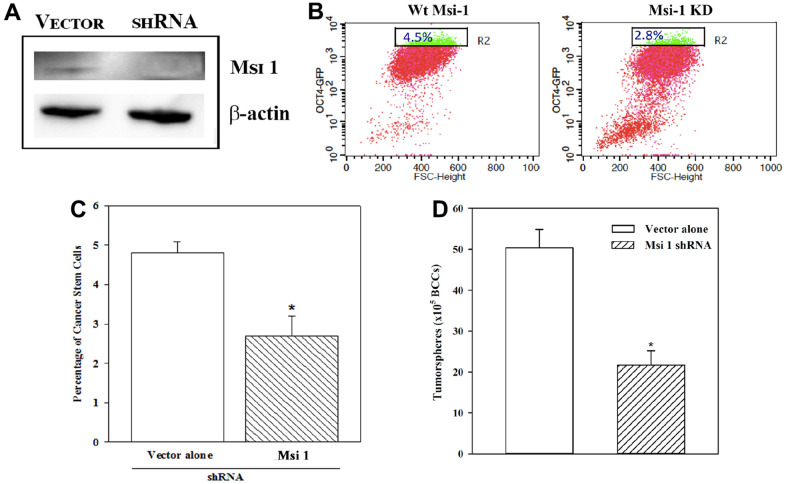
**CSCs in Msi-1 knockdown BCCs.** MDA-MB-231 with stable pOct4a-GFP was knockdown for Msi 1 (**A**) and then analyzed for Oct4a^hi^ cells (**B**). The mean±SD percentages of Oct4a^hi^ cells in the knockdown and vector (with scramble shRNA) transfected BCCs are presented for four different experiments (**C**). * *p*<0.05 vs. vector alone. The mean±SD percentages of tumorspheres for the Msi 1 knockdown and vector controls are presented for three different experiments (**D**). * *p*<0.05 vs. Msi 1 shRNA.

In this section, we describe a functional role for Msi 1 in CSC maintenance [8]. We knocked down Msi 1 in BCCs and then evaluated the percentage of CSCs, relative to vector transfectants. The latter comprised cells with the basic Msi 1 shRNA vector. Western showed a bright band for Msi 1 in the vector transfectants and a faint band when Msi 1 was knockdown, which validated its decrease (Figure 2A). Since Msi 1 knockdown BCCs stably expressed pOct4a-GFP, we applied GFP intensity to assess the percentage of Oct4a^hi^ cells. Flow cytometry indicated a marked decrease of Oct4a^hi^ BCCs in the Msi 1 knockdown cells as compared to vector transfectants (Figure 2B). A summary of biological and technical replicates indicated a significant (*p*<0.05) decrease of Oct4a^hi^ BCCs/CSCs in Msi 1 knockdown BCCs, as compared to vector transfectants (Figure 2C). These findings supported a functional role for Msi 1 in CSC maintenance.

We next studied Oct4a^hi^ Msi 1 knockdown BCCs and vector transfectants for tumorspheres. The number of tumorspheres/10^5^ cells were <50% in the Msi 1 knockdown cells as compared to vector transfectants (Figure 2D). In total, the results supported a role for Msi 1 in CSC maintenance. The Msi 1 knockdown BCCs failed serial passaging after the third transfer.

### Effects of Msi 1 on Hes1 and Oct4a reporter gene activity

As a RNA binding protein, Msi 1 can control the translation of genes, including those associated with multipotency [37]. Additionally, Msi 1 could regulate the expression of transcription factors that are required for stem cell maintenance [38]. In order to gain insight into a role of Msi 1 in stem cell regulation, we performed computational studies with specific stem cell associated gene sequences, as described [32]. Specifically, we investigated RNA-Protein interactome database to predict the interaction between Msi 1 and stem cell-associated proteins. Table 1 summarizes the predicted bidirectional interaction between known stem cell-associated proteins with mRNA associated with stem cell maintenance including Msi 1. Msi 1 is predicted to interact with the transcript of Oct4 and REST. Thus, as an RNA binding protein, Msi 1 would be able to regulate genes needed to maintain multipotency [8]. The predicted interactions indicated a complex network among Msi 1 and stem cell associated genes.

**Table 1:** Predicted interaction between stem cell associated proteins and target mRNA.

		**Binding proteins**			
		**Msi-1**	**REST**	**Oct4b**	**Oct4a**
**Target mRNA**	Oct4a	0.65	0.44	0.22	
	Oct4b	0.81	0.85		0.78
	REST	0.98		0.92	0.95
	Msi-1		0.62	0.25	0.32

Shown is a summary of the predicted probabilities for interactions between stem cell-associated proteins and target mRNA, based on RNA-Protein interaction (RPI)-Seq. The predicted values, 0.0 - 1.0, suggested > .5 to be statistically significant for potential interaction between the protein and target site. There is a positive relationship between the interaction value and the probability that the binding protein significantly interacts with the target mRNA. The data show a high probability that Msi 1 and Oct4 variant proteins bind to the REST mRNA, suggesting a possible mechanism of interaction or involvement with the REST pathway.

**Figure 3:**
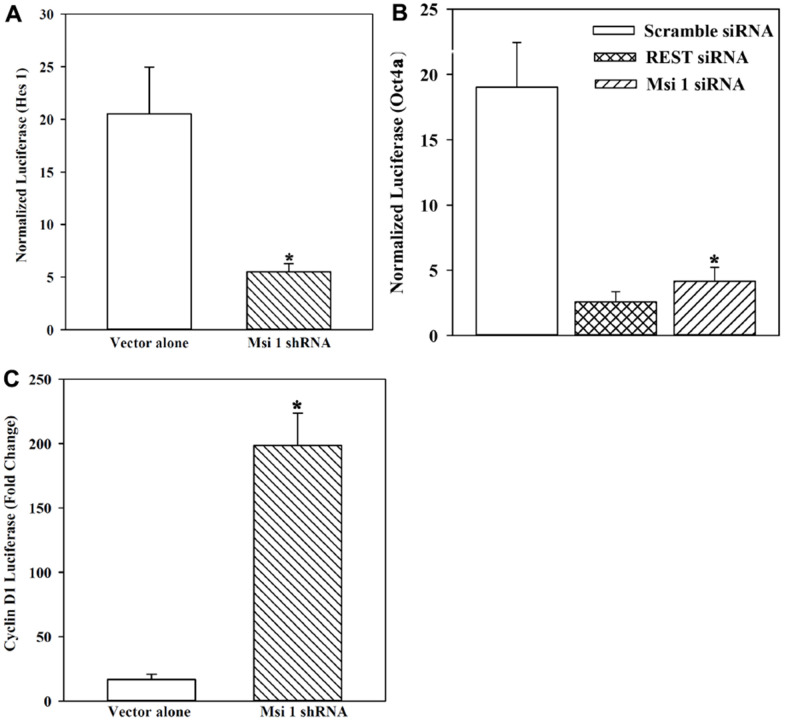
**Reporter gene activity under the control of the 5’ regulatory region of *Hes 1*, *Oct4a* and *Cyclin D1* in Msi 1 knockdown BCCs.** (**A**) BCCs, knockdown for Msi 1 or transfected with vector were studied for reporter gene in which luciferase was downstream of the *Hes 1* 5’ regulatory region. Normalized luciferase levels are expressed as fold changes over pGL3 transfectants. The data are presented as the mean±SD of four independent experiments, each in duplicate. * *p*<0.05 vs vector alone. (**B**) The studies in `B’ are repeated except for the reporter gene containing *Oct4a* 5’ regulatory region. REST siRNA served as a positive control. The data are presented as for `A’. Each experiment was performed in duplicate. * *p*<0.05 vs vector alone. (**C**) The studies in `B’ are repeated except for analyses for cyclin D1 activity and the data for three independent experiments with each performed in duplicate, presented as for `A’. * *p*<0.05 vs vector alone.

To test these computational findings, we transfected a reporter gene containing the 5′ regulatory regions of *Hes 1* in Msi 1 knockdown BCCs and compared with vector transfectants. *Hes 1* was selected because it is activated in stem cells and requires Msi 1 to activate Notch 1 for its transcription [39, 40]. Normalized luciferase, presented as fold change over basic pGL3-Luc transfectants, showed a 4-fold decrease in Msi 1 knockdown BCCs, relative to vector transfectants (Figure 3A).

Next, we asked if Msi 1 could regulate the key stem cell gene, *Oct4a*. We transfected pOct4a-Luc in BCCs, knockdown for Msi 1 (Figure 1B). We included REST as a control because it regulates stem cell and BCC growth [9, 41]. Luciferase level was significantly (*p*<0.05) decreased when Msi 1 and REST were knockdown as compared to scramble siRNA (Figure 3B). The results showed a role for Msi 1 in the regulation of key stem cell genes.

### Role of Msi 1 in Cyclin D1 reporter gene activity

CSCs are mostly cycling quiescence [24, 42]. Since Msi 1 knockdown reduced the relative percentage of CSC (Figure 2), this should correlate with cells transitioning to cycling. We studied Cyclin D1 activity with a reporter gene system in which luciferase was regulated by the 5’ regulatory region (Promoter) of C*yclin D1.* BCCs with Msi 1 knockdown or shRNA vector (vector alone) were transiently transfected with pCyclin D1-Luc or pGL3-Luc (control). Normalized luciferase was significantly higher in Msi 1 knockdown BCCs as compared to vector transfectants (Figure 3C). The findings indicated an increase in cyclin D1 activity when Msi 1 was knockdown.

### Cell senescence could not explain loss of stemness in Msi 1 knockdown BCCs

**Figure 4:**
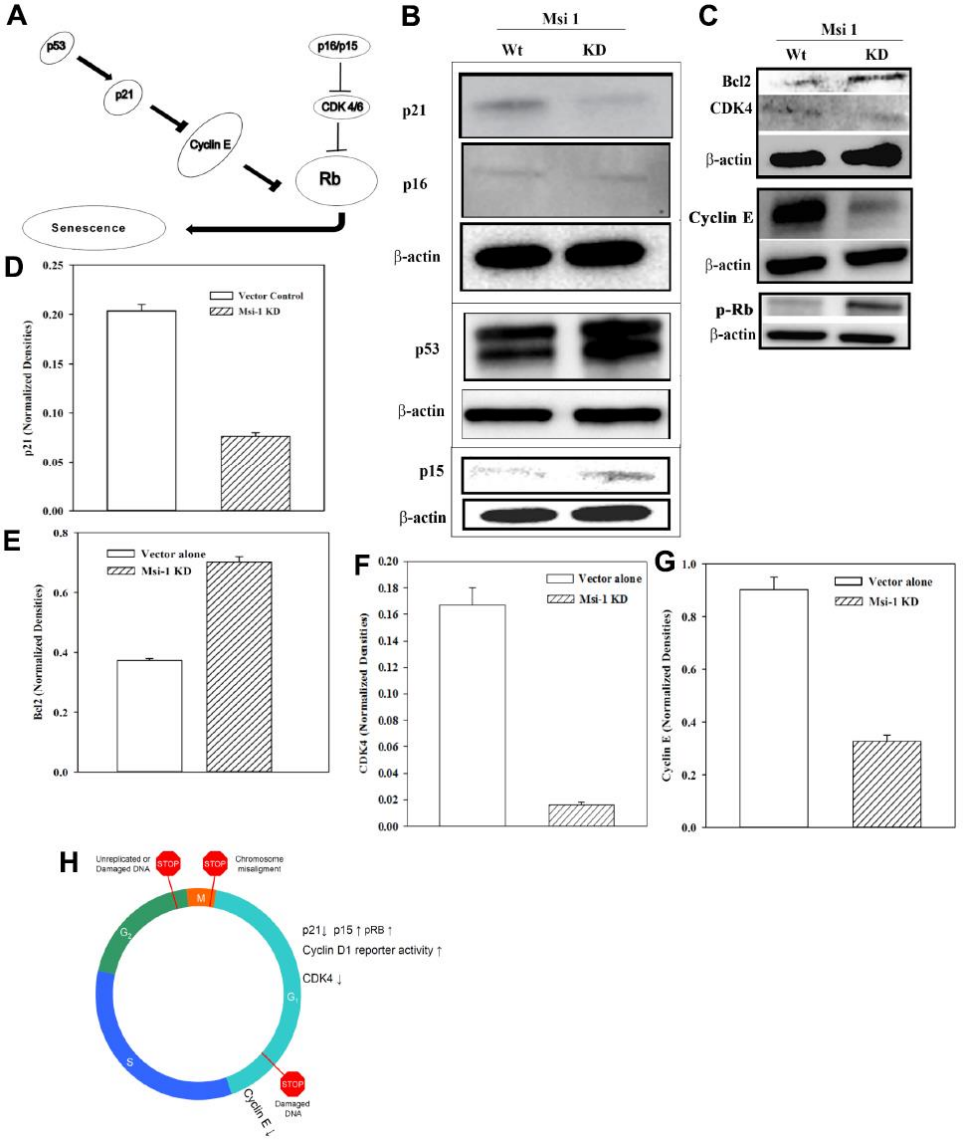
**Cell cycle-associated gene levels in Msi 1 knockdown BCCs.** (**A**) Shown is the pathway tested by Western blots. (**B**) Western blots were performed with whole cell extracts for p21, p16 in the same blot and separate blots for p15 and p53. (**C**) Western blots were performed for Bcl2 and CDK4 in the same blot and separate blots for Cyclin E and p-Rb. Each membrane was stripped and reprobed for β-actin. The blots represent the mean±SD of three independent experiments. (**D**–**G**) Normalized bands are shown for key proteins in the Western blots, ±SD, n=3. (**H**) A summary of the cycling protein levels relative to the cycling phase.

Msi 1 knockdown decreased the percentage of CSCs in BCCs (Figure 2). This correlated with reduced Oct4a and *Hes 1* reporter gene activities (Figure 3). Since passaging of Msi 1 knockdown BCCs in culture could not exceed 3 serial transfers, we asked if the decrease in CSCs could be due to cellular senescence. We addressed this question by testing the pathway shown in Figure 4A [43]. We performed Western blots for p53, p21 and p16 using whole cell extracts from MDA-MB-231, knockdown for Msi 1 (KD) or transfected with vector alone (wild type, Wt) (Figure 4B). The bands for p53 and p16 were similar. However, p21 was decreased with extracts from Msi 1 knockdown cells. The decrease in p21 could be linked to cell cycle transition, which was corroborated with an increase in cyclin D1 luciferase activity (Figure 3C).

Since the data showed no evidence of cell senescence, we analyzed the cell extracts for the anti-apoptotic Bcl2 [44]. The results showed increased Bcl2 in the Msi 1 knockdown BCCs (Figure 4C, top row). Thus, while the decrease in Msi 1 does not seem to cause cell senescence, reduced Msi 1 might lead to the BCCs compensating for survival through increased Bcl2.

### Cell cycle-promoting proteins in Msi 1 knockdown BCCs

Msi 1 knockdown BCCs led to reduced tumor growth *in vivo* [8]. However, this decrease could not be explained by cell cycle quiescence (Figures 3C, 4B). We therefore performed Western blots for CDK4 and Cyclin E with whole cell extracts from MDA-MB-231, knockdown for Msi 1 or transfected with vector alone (Figure 4C). Interestingly, p15, which can inhibit CDK4, were slightly increased in Msi 1 knockdown BCCs, which might explain the decreases in CDK4 and Cyclin E (Figure 4C). Taken together, the decrease in p21 with increased p-RB would allow for cyclin transition of BCCs. However, this increase could be moderately mitigated by increased p15 to blunt CDK4 and Cyclin E (Figure 4H).

### Msi 1 in PTEN-PI3K-AKT pathway

MDA-MB-231 cells comprise approximately 5% CSCs [24] (Figure 2). CSCs were significantly decreased in Msi 1 knockdown BCCs (Figure 2). This decrease could not be explained by senescence. Msi 1 knockdown BCCs were reported to have smaller tumor size [8]. However, this reduced growth could not fully explain the cycling status of the Msi 1 knockdown BCCs (Figure 4). It appeared that G1 transition might be mitigated by increases of other proteins to slow S-phase transition (Figures 3, 4). In this set of studies, we investigated if upstream pathways might explain the decrease of BCC growth in the Msi 1 knockdown BCCs.

**Figure 5:**
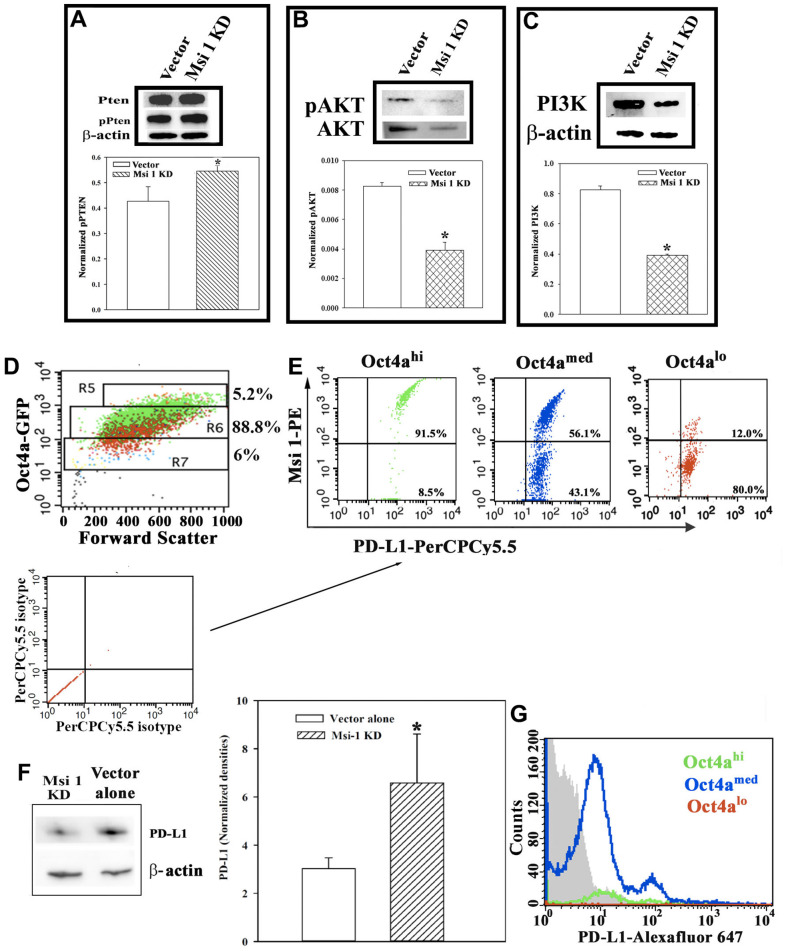
**PTEN-PI3K-AKT axis and PD-L1 expression in BCC subsets.** Western blots for PTEN and pPTEN (**A**), pAKT and AKT (**B**) and PI3K (**C**). Representative bands for the Western blots are shown with the mean±SD of normalized band densities for five biological replicates. (**D**) Gating strategy for BCC subsets were used for PD-L1 by flow cytometry. R5 represents Oct4^hi^; R6, Oct4a^med^; R7, Oct4a^lo^. The voltage was consistently applied to isolate the various BCC subsets. (**E**) The dot blots represent three different independent experiments to assess the expression of Msi-1 and/or PD-L1 in BCCs shown in `D’. The isotype for these studies are shown below with arrows pointing to the experimental plots. (**F**) Western blot for PD-L1 with extracts from Msi-1 KD BCCs or vector control. The mean normalized band densities for three independent Western blots are shown at right (±SD). (**G**) Flow cytometry was conducted for PD-L1 with Msi 1 knockdown BCCs. The cells were gated as depicted in `D’. The grey region depicts fluorescence of isotype control. The histogram represents three independent studies with the percentages of each subset stated in the quadrants. * *p*<0.05 vs vector transfectants.

We examined PTEN since this can be inhibited by Msi 1 [1]. PTEN activation could influence pAKT and PI3K levels. This would occur by p-PTEN blocking the activation of PI3K, which is required to activate AKT. We performed Western blots for PI3K, total AKT, p-AKT, total PTEN and p-PTEN with whole cell extracts from MDA-MB-231, knockdown for Msi 1 or transfected with vector. p-PTEN level was significantly (*p*<0.05) increased in the knockdown as compared to the vector-transfected BCCs (Figure 5A). This increase correlated with significant (*p*<0.05) decreases in pAKT and PI3K in the Msi 1 KD BCCs, as compared to vector transfectants (Figures 5B, 5C). Overall, the results showed an increase pPTEN and decreases in pAKT and PI3K, consistent with reduced tumor growth.

### Msi 1 and PD-L1

Triple negative BCCs express high levels of the T-cell inhibitory molecule, PD-L1, which is a target for immune checkpoint inhibitors [25]. PD-L1 expression on triple negative BCCs depended on decreased PTEN and increased PI3K [25]. However, we noted an increase in p-PTEN in Msi 1 knockdown cells with concomitant decreases in p-AKT and PI3K (Figures 5A–5C). We first examined wild-type BCCs for PD-L1 expression. We used the gating scheme shown in Figure 5D to analyze different BCCs for Msi 1 and PD-L1. The voltage used to isolate BCC subset has been consistent and therefore served as an internal control [24]. Thus, if a particular subset seems higher, this is due to the total number of events since the relative percentages of cells remain consistent with the literature [24]. Both proteins were expressed in Oct4a^hi^ BCCs, which are predominantly CSCs (Figure 5E- left panel, upper right quadrant). We also noted a small subset within the Oct4a^hi^ BCCs that express PD-L1 but were negative for Msi 1 (lower right quadrant). This pattern was similar for Oct4a^med^ BCCs, except for a higher subset expressing only PD-L1 (Figure 5E, middle panel). In contrast, the majority of Oct4a^lo^ BCCs express PD-L1 alone (Figure 5E, right panel). In summary, most of Oct4a^hi^/CSCs co-expressed PD-L1 and Msi-1 whereas the less mature BCC subsets mostly express PD-L1 alone, but do not coexpress Msi-1.

### PD-L1 expression on Msi 1 KD BCCs

Figure 5D, 5E show a partial correlation between PD-L1 and Msi 1. We therefore asked if Msi 1 regulates PD-L1 expression in BCCs. We first performed Western blot for PD-L1 in Msi 1 knockdown BCCs, and BCCs transfected with vector alone. The results showed significantly lower levels of PD-L1 when Msi 1 was knocked down in heterogeneous BCCs (Figure 5F). Next, we determined if the decreased PD-L1 in the Msi 1 knockdown BCCs could be linked to a particular BCC subset. We performed flow cytometry for PD-L1 in Msi 1 knockdown BCCs. Since the BCCs also expressed pOCt4a-GFP, we gated the three main BCC subsets, Oct4a^hi^, Oct4a^med^ and Oct4a^lo^. The vertical line demarcated the fluorescence for isotype at left. We showed two populations of Oct4a^med^ BCCs based on the MFI (Figure 5G, blue histogram). These two subsets are consistent with two populations of PD-L1 expressing cells in the heterogeneous BCCs (Figure 5E, middle panel). The MFI for CSCs was similar to isotype control (Figure 5G, green histogram). Similarly, it was reduced to almost undetectable Oct4a^lo^ BCCs (Figure 5G, red histogram). Together, these studies suggested Msi 1 knockdown affected PD-L1 expression on the BCC subsets. The CSCs and Oct4a^lo^ BCCs were mostly affected with respect to PD-L1 expression.

## DISCUSSION

We demonstrated increased Msi 1 in CSCs, as compared to the other BCC subsets within the hierarchy of BCCs (Figure 1) [31]. The increased levels of Msi 1 on CSCs were key to maintaining multipotency. This was demonstrated by significant decrease of CSCs when Msi 1 was knockdown with concomitant increase in the more mature BCCs, which suggested differentiation of CSCs (Figure 2B, 2C). The reduced frequency of CSCs correlated with a significant decrease in tumorspheres in the Msi 1 knockdown BCCs (Figure 2D). The decrease in CSCs could not be explained by cell senescence or cell death (Figure 4). We surmised that the decrease could be due to an increase in differentiation since the knockdown cells showed a higher frequency of Oct4a^lo^ BCCs (Figure 2B, right panel). The increase in the more mature Oct4a^lo^ BCCs indicated differentiation of CSCS. The comparison between CSCs (Oct4a^hi^) and Oct4a^lo^ BCCs is appropriate because in the analyses, we acquired similar number of cells.

The knockdown of Msi 1 did not totally ablate CSCs (Figure 2B). This could be due to the small remaining Msi 1 since this is not knockout but knockdown Msi. On the other hand, there are documented cases of dedifferentiation of BCCs via cell autonomous method [45]. However, the evidence indicated that decreased Msi 1 induces differentiation since the other BCC subsets were increased (Figure 2).

We conducted molecular studies to understand how Msi 1 supported CSCs with reporter gene studies [8]. Luciferase under the control of the 5′ regulatory regions of stem cell-linked genes, *Hes 1* and *Oct4a*, indicated that Msi 1 is involved in their expression (Figure 3) [40, 41]*.* These findings indicated that Msi 1 controls the expression of multipotent-associated genes, specifically, Oct4a, REST and *Hes 1*. The strong link between Msi 1 and stem cell genes was further enhanced with computational studies (Table 1). The loss of stemness, combined with increased Oct4a^lo^ BCC subsets in the Msi 1 knockdown BCCs, correlated with an increase in *Cyclin D1* reporter gene activity (Figure 3C). This indicated that decreased Msi 1 could induce CSCs to differentiate and to proliferate. However, there are other mechanisms to deter complete cycling transition, as outlined in the next paragraph.

Msi 1 knockdown BCCs grew slowly as compared to vehicle transfectants [8]. These knockdown BCCs could be serially passaged after three transfers, which is in line with a significant loss of CSCs (Figure 2). However, the reduced growth was contrary to decreased p21 (Figure 4). We eliminated cell senescence in the Msi 1 knockdown BCCs by showing a decrease of p16 (Figure 4D). Rather, we noted an increase in Bcl2 that could facilitate the survival of Msi 1 knockdown BCCs. (Figure 4C). Despite the cyclin proteins indicating slow cycling, reporter gene studies showed an increase in Cyclin D1 activity, which is needed for G1 transition (Figure 3). Insights into the cycling behavior of the Msi 1 knockdown BCCs could be explained by enhanced p15 to decrease CDK4 (Figure 4). Further halting of cycling could occur by decreased Cyclin E to slow the transition at S-phase (Figure 4H). Reduced pAKT and PI3K with concomitant p-PTEN could also explain the reduced growth (Figure 5).

In order to consider how the findings could be translated to patients, it is prudent to examine how a small molecule that inhibits Msi 1 might increase pPTEN. The downstream changes could be significant considering the high levels of PD-L1 in CSCs, which was decreased with Msi 1 knockdown (Figure 5). PD-L1 was increased in the Oct4a^med^ and Oct4a^lo^ BCCs, despite less Msi 1 (Figures 1, 5E). Msi 1 knockdown in unsorted/heterogeneous BCCs led to decreased PD-L1 by Western blot (Figure 5F). Stratification of BCCs indicated low to undetectable PD-L1 on CSCs with minimum detection on Oct4a^lo^ BCCs. We observed two populations of Oct4a^med^ BCCs – PD-L1 with Msi 1 and PD-L1 without Msi 1 (Figure 5E, middle panel). In the Msi 1 knockdown BCCs, we continued to observe two populations of Oct4a^med^ BCCs with different PD-L1 levels, albeit reduced expression. The data could not link Cyclin D with PD-L1 since the former was conducted to prove cell cycle exit from the otherwise quiescent CSCs. Since we observed a loss of PD-L1 on Msi 1 knockdown CSCs, it is unlikely that this cyclin could be associated with PD-L1 expression. It is therefore important that future studies conduct omics studies with Msi 1 knockdown BCCs, in particular single cell analysis. The Oct4a^med^ BCCs are close to CSCs with respect to cell maturity and their behavior could be important to predict chemoresistance as well as ease of dedifferentiating to CSCs [31]. We observed an interesting finding in which the Oct4a^lo^ BCCs showed almost undetectable PD-L1 (Figure 5G). This finding was interesting because it is this subset that could be easily dedifferentiated by the secretome from cells of the bone marrow niche to CSCs [42].

In general, the results are consistent with loss of stemness and cycling quiescence in the Msi 1 knockdown cells and this correlated with an increase of G1 phase of the cell cycle [24, 42]. Since cellular dormant BCCs adapt properties of CSCs in the bone marrow, this study has implications for treatment to prevent BCCs converting to dormancy and to incorporate a treatment to reverse and eliminate the quiescent dormant CSCs [24, 45, 46]. Despite the identification of intercellular communication between CSCs and bone marrow niche cells through connexin 43, targeting these connections is not an attractive option since similar connection is required in hematopoietic regulation [24, 47, 48]. Recent studies suggested N-cadherin as a target for dormant BCCs in the bone marrow [23]. Despite Msi 1 link to stem cell genes such as Notch 1, small molecules that inhibit this RNA binding protein could be a new method to combine with other drugs for BC treatment. This is particularly important because BCCs could adapt dormancy at any time during the course of the disease as well as during treatment. Thus, Msi 1 could be added to the current information to leverage targeted treatment to eliminate and to prevent dormancy of BCCs in the bone marrow [48].

We validated the link between Msi 1 and PD-L1 in CSCs based on significant reduction of CSCs following Msi 1 knockdown. This study has just begun to study this link, which is fundamental to the treatment of CSCs, perhaps by combining immune therapy with other treatments. CSCs are difficult to treat due to increased drug resistance genes and cycling quiescence. Thus, other avenues of treatment are necessary. The stem cell genes such as BMI and Notch have failed to have an impact on solid tumors. This could be due to the dose limit of drugs, which could be toxic to the endogenous stem cells. This is particularly relevant to bone marrow, which is the home of hematopoietic stem cells.

Historically, dormant breast cancer cells have been shown to survive for decades [49]. This can occur by a method in which the BCCs are able to evade the immune system [50]. Several methods have emerged to explain how CSCs could evade the immune system. These include increased interaction with M2 macrophages and regulatory T-cells, which are mostly facilitated by the tissue microenvironment such as mesenchymal stem cells in bone marrow [50–52]. There are other emerging studies to link molecular pathways in CSCs to maintain PD-L1 [53]. This study now links Msi 1 to PD-L1. Since Msi 1 is an RNA binding protein that can compete with miRNA, future studies are in the process of determining the RNA targets of Msi 1 [8]. Data from these studies will provide insights on the role of Msi 1 in regulating PD-L1 on CSCs.

**Figure 6:**
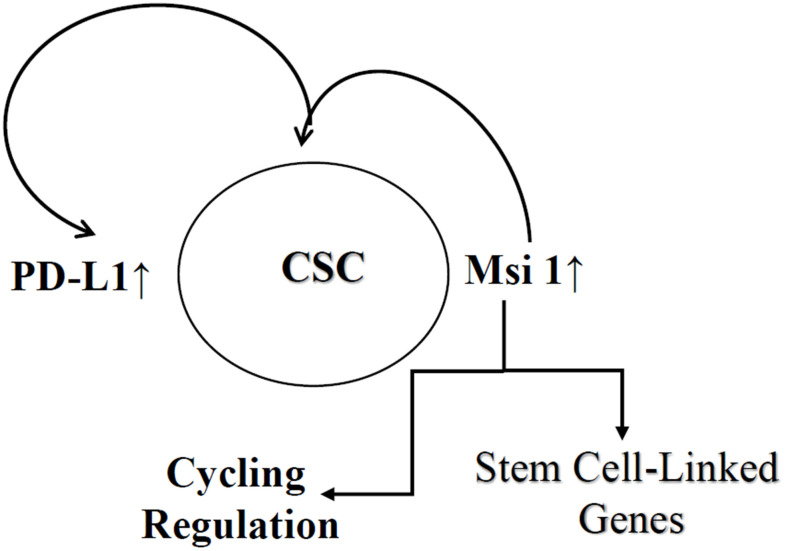
**Key summary of findings.** Shown is increased expression of PD-L1 and Msi 1 in CSCs. Msi 1 supports CSC partly by regulating stem cell associated genes and also modulate PD-L1 expression. The sum of these regulatory mechanism leads to immune protection of CSCs and cycling regulation.

Overall, the focus on CSCs allowed us to extrapolate on how this report could be included in the literature on BC dormancy in the bone marrow (Figure 6) [54, 55]. The studies showed increased PD-L1 and Msi 1 on CSCs. Mesenchymal stem cells are major niche cells in bone marrow and their interaction with CSCs will increase regulatory T-cells to protect the cancer cells [56]. The question is what role PD-L1 has in protecting the CSCs from immune response in the bone marrow during the dormant phase. Since the frequency of CSCs is low, the findings in this study showed that during molecular stratification of tumors, testing a bulk tumor could show negative phenotype for PD-L1. This could eliminate patients from treatment with specific immune checkpoint inhibitor. This statement is based on the relatively small population of CSCs, making it a challenge to detect for Msi 1. In this regard, missing this relatively small subset could be detrimental to eliminate the population of BCCs that could be responsible for cancer resurgence.

## Supplementary Material

Supplementary Figures
