# Supplementary material for: Increased expression of musashi 1 on breast cancer cells has implication to understand dormancy and survival in bone marrow
Source: Aging (Albany NY). 2023 Mar 29;15(9):3230–48. doi: 10.18632/aging.204620 (PMC10449290; doi:10.18632/aging.204620)
Supplement: Supplementary Figures [file aging-15-204620-s001.pdf]

SUPPLEMENTARY FIGURES

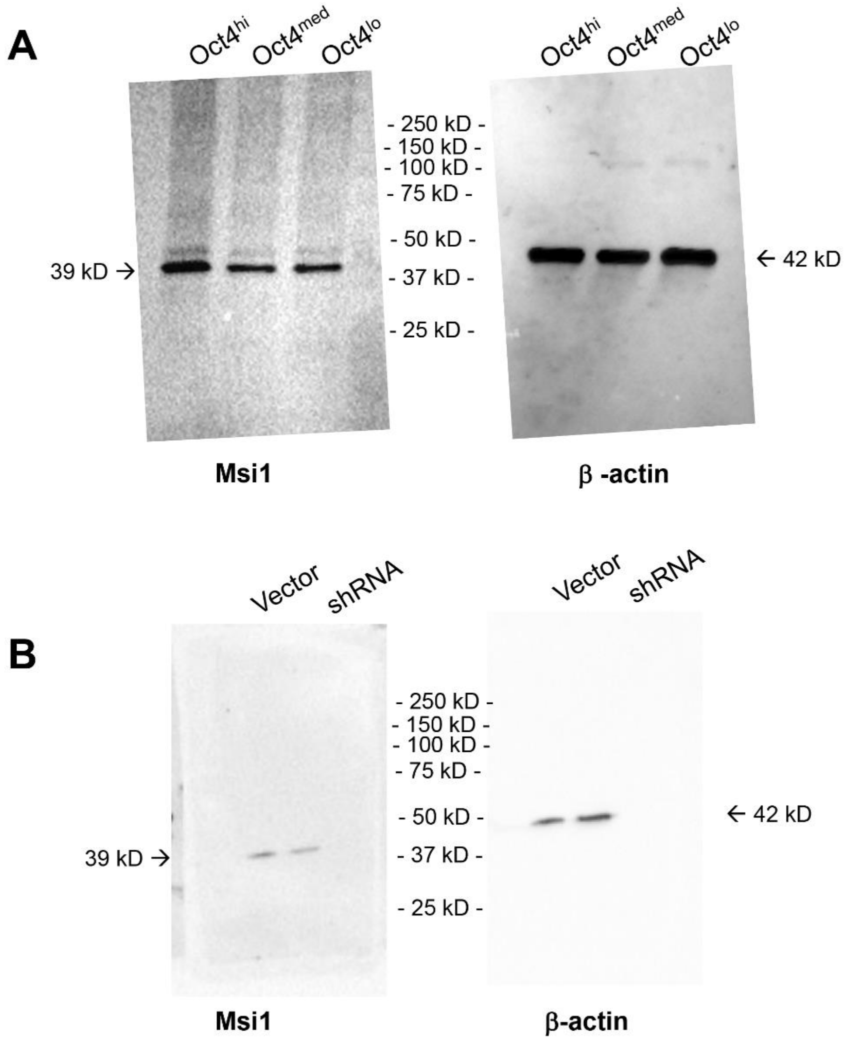

Supplementary Figure 1. Western blotting evaluation of Msi 1 in MDA-MB-231 cells. Western blotting was performed using anti-Msi-1 (Abcam, Cambridge, MA) 1:1000 in (**A**) Oct4^{hi}, Oct4^{med}, and Oct4^{lo} MDA-MB-231 cells and (**B**) Msi-1 knockdown MDA-MB-231 cells. Anti-β-actin (Sigma-Aldrich) 1:10000 was used as a housekeeping gene. Highest levels of Msi1 were observed in the Oct4^{hi} subpopulation of MDA-MB-231 cells, and Msi 1 was decreased in the knockdown cells.

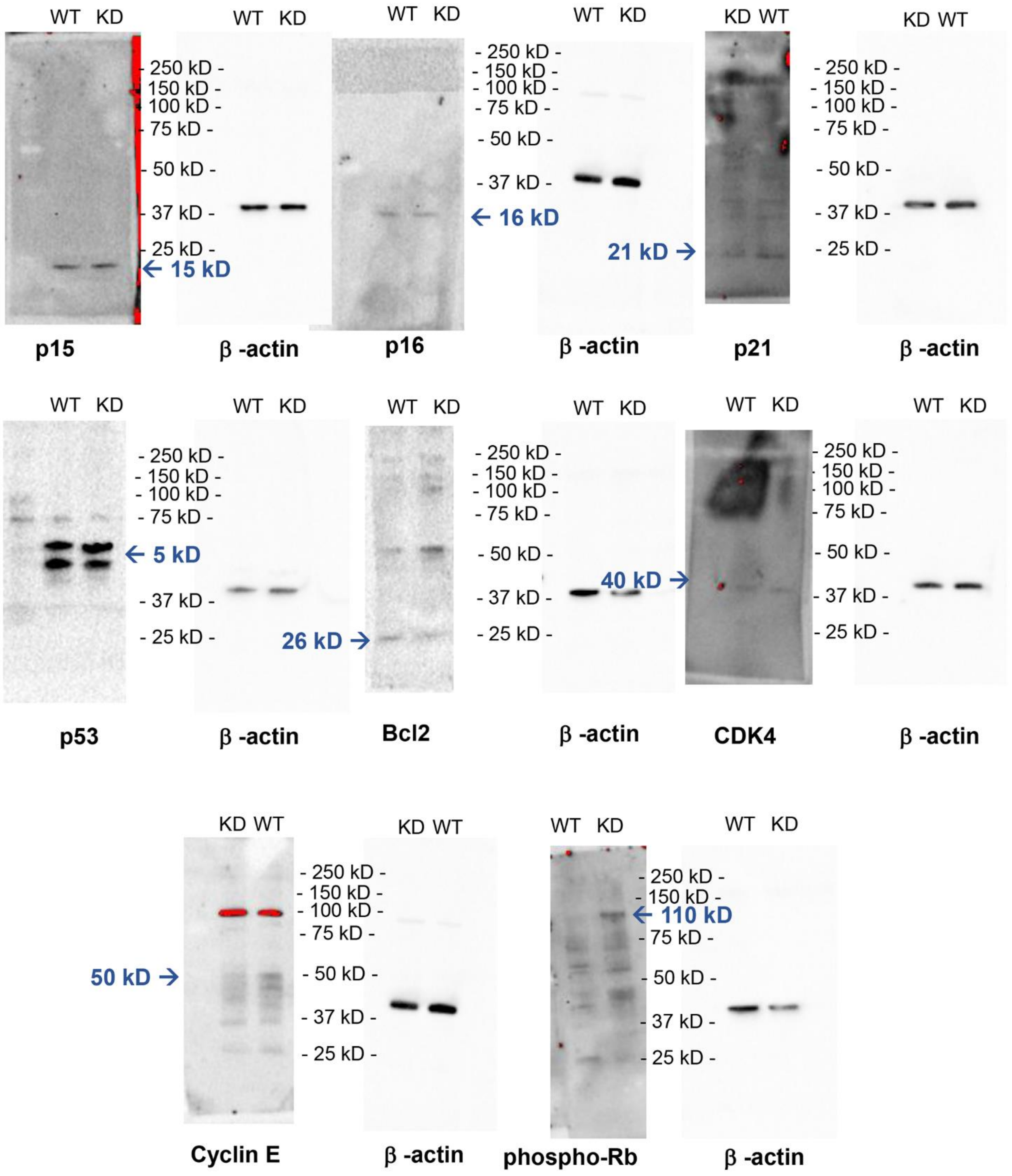

Supplementary Figure 2. Western blotting evaluation of cell cycle related genes. Western blotting was performed of wild type (WT) and Msi 1 knockdown (KD) MDA-MB-231 cells using anti-p15 (Santa Cruz Biotechnology Inc, Santa Cruz, CA) 1:1000, anti-p16 (Cell Signaling Technology, MA) 1:1000, anti-p21 (R&D Systems, Minneapolis, MN) 1:1000, anti-p53 (Santa Cruz Biotechnology Inc, Santa Cruz, CA) 1:1000, anti-Bcl2 (Santa Cruz Biotechnology Inc, Santa Cruz, CA) 1:1000, anti-CDK4 (Santa Cruz Biotechnology Inc, Santa Cruz, CA) 1:1000, anti-Cyclin E (Cell Signaling Technology, Massachusetts, USA) 1:1000, anti-phospho-Rb (Cell Signaling Technology, Massachusetts USA) 1:1000. Anti-β-actin (Sigma-Aldrich) 1:10000 was used as a housekeeping gene. All β-actin bands were observed at the expected 42 kD molecular weight.

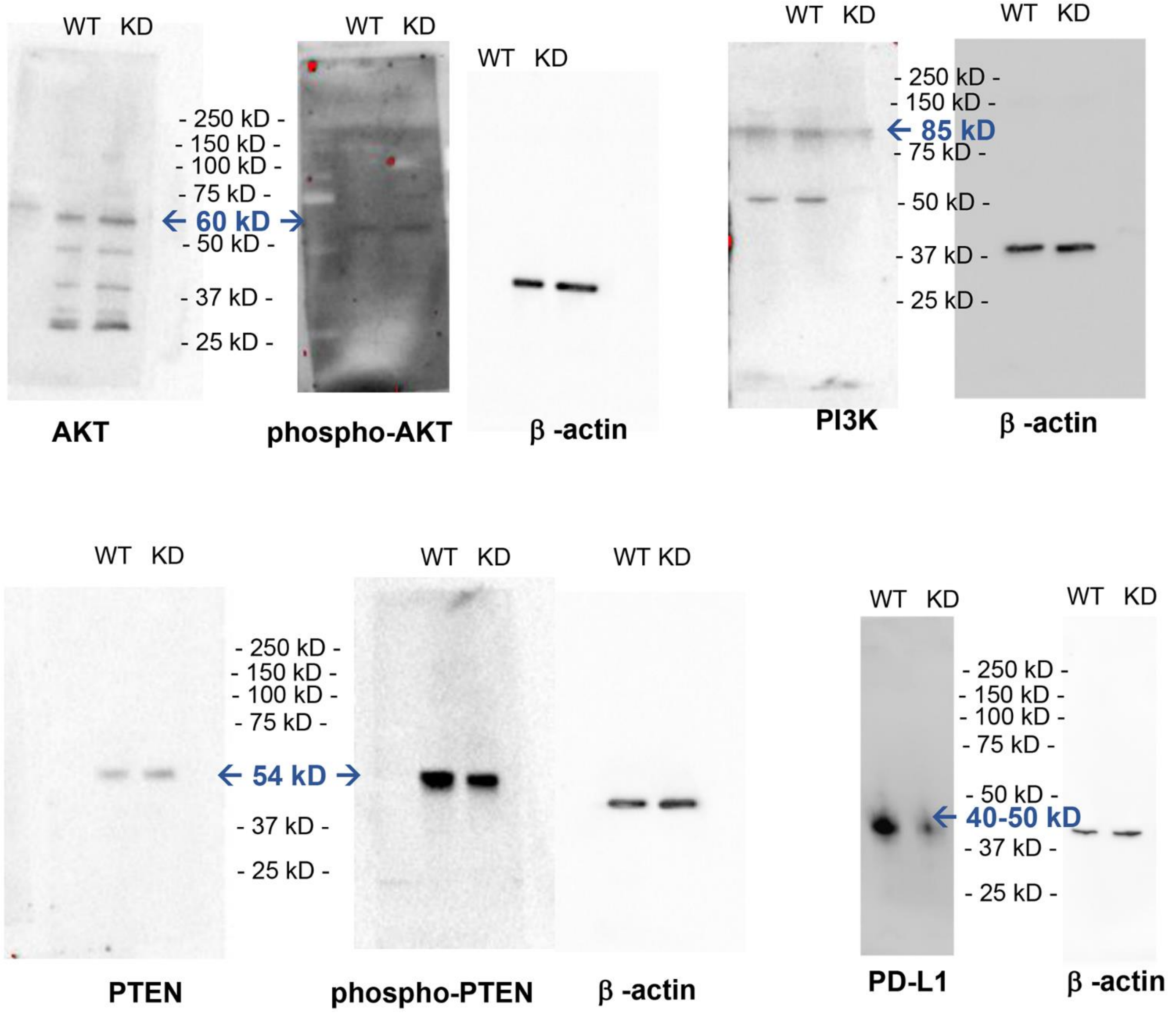

Supplementary Figure 3. Western blotting evaluation of PTEN-PI3K-AKT axis and PD-L1. Western blotting was performed of wild type (WT) and Msi1 knockdown (KD) MDA-MB-231 cells using anti-PTEN and anti-phospho-PTEN (Cell Signaling Technology, MA) 1:1000, anti-AKT and anti-phospho-AKT (Cell Signaling Technology MA) 1:1000, and PI3K (Millipore, St. Louis, MO), and anti-PDL1 (Cell Signaling Technology MA) 1:1000. Anti-β-actin (Sigma-Aldrich) 1:10000 was used as a housekeeping gene. PTEN and phospho-PTEN were normalized to phospho-PTEN and phospho-AKT, respectively.
